# Nashville Journal of Medicine and Surgery

**Published:** 1851-04

**Authors:** 


NASHVILLE JOURNAL OF MEDICINE AND SURGERY.

This is the title of a new Medical Journal, the publication of which has just commenced at Nashville, Tenn. It is edited by W. K. Bowling, M. D., Professor of the Institutes and Practice of Medicine in the Medical Department of the University of Nashville.

We cordially welcome Dr. Bowling to the editorial corps, and wish him abundant prosperity in his new enterprise. If the medical profession are true to their best interests they will secure success to the Nashville Journal of Medicine and Surgery. Everything that keeps the busy practitioner of medicine in communication with the active minds of the profession should be looked upon as an object worthy of favor. We should say more on this subject, if our waning space did not forbid.

